# Feasibility of a laboratory-based accelerometer calibration protocol for children with intellectual disabilities

**DOI:** 10.1186/s40814-015-0014-2

**Published:** 2015-05-24

**Authors:** Arlene M. McGarty, Victoria Penpraze, Craig A. Melville

**Affiliations:** 1 Institute of Health and Wellbeing, College of Medical, Veterinary and Life Sciences, University of Glasgow, Gartnavel Royal Hospital, 1055 Great Western Road, Glasgow, G12 0XH Scotland UK; 2 School of Life Sciences, College of Medical, Veterinary and Life Sciences, University of Glasgow, Glasgow, Scotland UK

**Keywords:** Physical activity, Accelerometer, Calibration, Intellectual disabilities, Children

## Abstract

**Background:**

Accelerometry has not been calibrated for the estimation of physical activity in children with intellectual disabilities (ID), raising questions regarding the validity of interpreting accelerometer data in this population. Various protocols and criterion measures have been used in calibration studies involving typically developing (TD) children; however, the suitability of these activities and measures for children with ID is unknown. Therefore, this study aimed to test the feasibility of a laboratory-based calibration protocol for children with ID. Specifically, the feasibility of activities, measurements, and recruitment was investigated.

**Methods:**

Five children with mild to moderate ID (10.20 ± .98 years) and a comparative sample of five TD children (12.40 ± .01 years) participated in this study. Participants performed a free-living and treadmill-based activity protocol during two laboratory-based sessions. Activities were performed for 5 min and ranged from sedentary to vigorous intensity. Treadmill activities ranged from 3 to 8 km/h, and free-living activities included watching a DVD, passing a football, and jumping jacks. Resting energy expenditure was measured, and a graded exercise test was used to assess cardiorespiratory fitness. Breath-by-breath respiratory gas exchange and accelerometry were continually measured during all activities. Feasibility was assessed using observations, activity completion rates, and respiratory data.

**Results:**

All TD participants and one participant with ID completed the protocol. The physical demands of the treadmill activities affected the completion rate for participants with ID. No participant met the maximal criteria for the graded exercise test or attained a steady state during the resting measurements. Limitations were identified with the usability of respiratory gas exchange equipment and the validity of measurements. The school-based recruitment strategy was not effective, with a participation rate of 6 %. A significant (*z* = 13.21, *p* < .0001) difference in the relationship of $\dot{\mathrm{V}}{\mathrm{O}}_2$ and accelerometry was identified between ID and TD participants.

**Conclusions:**

Due to issues with the usability and validity of breath-by-breath respiratory gas exchange and recruitment, a laboratory-based calibration protocol is currently not feasible for children with ID. An alternative field-based protocol with a non-invasive criterion measure should be considered for future studies.

## Background

Accelerometers are a widely used objective measure that enables the quantification of physical activity levels. Accelerometers measure raw acceleration of the body in gravitational units (g), which is converted into an arbitrary unit (count). Counts can then be used to estimate physical activity, such as energy expenditure or time spent in moderate to vigorous intensity, through the application of prediction equations or cut points. These cut points and equations are developed by calibrating activity counts against a known biological measure, which is a form of validity-based research referred to as “value calibration” [1].

However, there is limited research investigating the validity and reliability of accelerometers for the estimation physical activity in children with ID [2]. This is impacting on the ability of researchers to accurately measure physical activity levels within this population [3]. Accelerometer counts have previously been validated in children with ID [4, 5], although no calibration studies have been conducted. To ensure the validity of measurements in children with ID, the development of population-specific cut points is urgently needed.

Calibration is a complex process, and there are many challenges in deriving a biological meaning from a raw biomechanical measure of acceleration. Furthermore, additional difficulties face calibration involving children, due to the relationship between energy expenditure and body mass and the influence of maturation [6]. In a review of accelerometer calibration in children, Freedson et al. [6] suggested that the development of population-specific cut points could limit the effect of these biological differences.

When differences between typically developing (TD) children and children with ID are considered, the validity of generalizing cut points is questionable [7]. For example, children with ID have lower reported levels of cardiorespiratory fitness than their TD peers [8–10]. Due to the effect that cardiorespiratory fitness has on energy expenditure and oxygen uptake ($\dot{\mathrm{V}}{\mathrm{O}}_2$) during activity, the generalizing of cut points calibrated in a population with higher fitness could lead to an underestimation or misclassification of activity intensity for a population with lower fitness. Due to validity issues such as these, Freedson et al. [11] discussed the importance of investigating and classifying fitness for calibration studies, which will provide information on relative activity and health.

The protocol of a calibration study fundamentally involves the concurrent measurement of accelerometry and a gold standard measure of activity. The use of a biological criterion measure of energy expenditure, specifically indirect calorimetry measured through respiratory gas exchange, is deemed most appropriate and has been widely used in studies involving children [12]. A laboratory-based protocol should include at least six free-living and/or treadmill-based activities which are representative of activities conducted by the study population [1, 2]. Previous calibration studies have employed a wide range of activities, including treadmill speeds ranging from 4 to 10 km/h, watching a DVD, playing catch, hopscotch, basketball dribbling, and martial arts exercises [13–15]. However, no standardized guidelines exist for the type of activities to be conducted or the amount of time for which these activities should be conducted.

To ensure the appropriateness of a calibration protocol for children with ID, the feasibility of the methods of measurement and the activity protocol should initially be tested. Respiratory gas exchange is not a measurement which has been widely conducted in children with ID. Furthermore, many of the activities used in previous studies involve sport-specific skills, co-ordination, and concentration and therefore may not be suitable for this population. Similarly, Oortwijn et al. [16] tested the feasibility of calibration protocol in a whole-room calorimeter in a sample of five children.

The aims of this study are to 1) test the feasibility of recruiting children from additional support needs (ASN) schools to a laboratory-based study, 2) test the feasibility of activities and measures, 3) test the feasibility of using breath-by-breath respiratory gas exchange, and 4) compare the relationship between accelerometry and $\dot{\mathrm{V}}{\mathrm{O}}_2$ between ID and TD participants.

## Methods

### Ethical consideration

This study was approved by the Medical, Veterinary, and Life Sciences College Ethics Committee, University of Glasgow. Written informed consent was obtained from both the participants and parents.

### Protocol

This study was conducted in three phases over two laboratory sessions: 1) familiarization, 2) preparation, and 3) data collection. The familiarization and preparation phases were conducted during session one. During the familiarization phase, the participant was introduced to the laboratory environment, with the aim of reducing participant anxiety in the data collection phase. The preparation phase allowed the participant to become familiar with the equipment and procedures and to ensure they were physically capable of safely completing the protocol. Data collection was conducted over the two sessions and consisted of anthropometric and resting energy expenditure (REE) measurements, treadmill-based and free-living activities, and a graded exercise test. Only participants with ID completed the REE measurements. Throughout the protocol, activity was measured using accelerometry and $\dot{\mathrm{V}}{\mathrm{O}}_2$ measured through breath-by-breath respiratory gas exchange.

The protocol used within this study is described in Table 1. The activity protocols of previous laboratory-based calibration studies in TD children, which included both treadmill and free-living activities, informed the development of the present protocol (Appendix). The activities chosen ranged from sedentary to vigorous intensity, were treadmill-based and free-living, and were skill and non-skill specific. This variety and number of included activities will enable the most appropriate and effective activities to be included in a full-scale calibration study.

**Table 1:** Protocol of measurements and activities performed

Activity	Description
Session 1	
Rest	Sitting in reclined position watching DVD
Treadmill-based activities	
Light intensity	
3 km/h	Walking at 3 km/h at zero gradient
Moderate intensity	
6 km/h	Jogging at 6 km/h at zero gradient
5 km/h at 5 %	Walking briskly at 5 km/h at 5 % gradient
Vigorous intensity	
8 km/h	Running at 8 km/h at zero gradient
Session 2	
Free-living activities	
Sedentary	
Sitting playing computer game	Sitting playing handheld Nintendo DS
Watching DVD	Sitting watching DVD
Drawing	Sitting drawing
Light intensity	
Passing football	Passing a football with a researcher
Playing catch	Standing throwing/catching a ball with a researcher
Standing playing computer game	Standing playing handheld Nintendo DS
Moderate intensity	
Step aerobics	Continual stepping on and off aerobic step
Hula hoop	Continual twirling of hula hoop around the waist
Interactive computer game	Playing interactive bowling on an Xbox Kinect
Vigorous intensity	
Jumping jacks	Continual jumping jacks/star jumps
Graded exercise test	Treadmill-based incremental fitness test

### Recruitment strategy

Participants with ID were recruited from ASN schools in Glasgow, Scotland. A researcher visited two schools, explained the study to children, and handed out information packs. If children were interested in participating, parents were asked to return a parent and child consent form to the researcher to allow discussion regarding participation. A convenience sample of TD children was recruited from the Glasgow area. All participant and parent travel expenses were reimbursed. Furthermore, children received a £30 high street voucher after completion of the study.

The exclusion criteria for participation were as follows: i) having a physical disability, ii) being non-ambulatory, or iii) being outwith the age range of 8–14 years.

### Measures

#### Anthropometric

Height was measured to the nearest 0.1 cm using a stadiometer (Seca Scales, Hamburg, Germany), and weight was measured to the nearest 0.1 kg using digital scales (Seca Scales, Hamburg, Germany). Measurements were conducted twice to produce a mean value whilst participants were wearing light clothing and no shoes.

#### Resting energy expenditure

Respiratory gas exchange was measured continually for 15 min to allow REE to be established. Throughout this measurement, participants sat in a reclined position and watched an age-appropriate DVD.

#### Activities

Participants were asked to complete four treadmill and ten free-living activities, each for 5 min (Table 1). These types of activities have been extensively conducted in calibration studies involving TD children [12] and were based on previously defined intensity classifications [15]. Prior to the activities, participants completed a 2-min treadmill-based warm-up. Additionally, rest periods were given between activities to allow measurements to return to within a resting range.

### Graded exercise test

Participants walked on the treadmill at a constant and self-selected pace. The gradient was increased from zero in increments of 2.5 % every 2 min until the participant was unable to continue. Concurrent with previous protocols in adolescents with ID [17], walking speed was self-determined. The use of self-determined pace for exercise testing in individuals with disabilities may prevent the test being discontinued due to participant anxiety [18].

The primary criterion for the attainment of maximal oxygen consumption (${{\dot{\mathrm{V}}\mathrm{O}}_2}_{\max }$) was a plateau in $\dot{\mathrm{V}}{\mathrm{O}}_2$ with increased workload. A secondary criteria of an increased respiratory exchange ratio (RER) >1.0 and a heart rate within 10 bpm of an age-adjusted estimate of maximal heart rate were also used [19, 20]. Predicted maximal heart rate was calculated using the equation: maximal heart rate = 220 − age.

### Instrumentation

#### Accelerometry

The ActiGraph wGT3X+ (ActiGraph LLC, Pensacola, FL, USA) is a small triaxial accelerometer which measures acceleration of the body across the vertical, horizontal, and perpendicular axes during movement. Prior to the session, the accelerometers were initialized in accordance with manufacturer specifications. The device was worn around the waist, positioned at the hip (at the iliac crest). In line with manufacturer guidelines for use in children, the device was attached using an elastic belt.

#### Heart rate

Heart rate was measured using a chest-worn heart rate monitor (Vantage, Polar Electro). The sensor was attached directly to the skin using an elastic belt, and measurements (beats per minute (bpm)) were recorded on the device receiver which was held by the researcher. Heart rate was recorded every minute during the graded exercise test and at the termination of the test.

#### Respiratory gas exchange

Respiratory gas exchange was measured using the Ultima CPX (Medical Graphics, MN, USA) which analyses expired gases on a breath-by-breath basis. Prior to each test, airflow, ventilatory volume, and gas analysers were calibrated using standard measures in accordance with manufacture guidelines. Participants wore a preVent (Medical Graphics, MN, USA) material mask which covered their nose and mouth. This was attached directly to a bidirectional flow meter, a sampling line, and measurement sensor. Data were initially recorded using standard threshold settings of minimum 50 mL $\dot{\mathrm{V}}{\mathrm{O}}_2$ and carbon dioxide production ($\dot{\mathrm{V}}{\mathrm{CO}}_2$), minimum 180 mL tidal volume, and RER between 0.5 and 2.6. These standard threshold settings are specific to the Ultima CPX system and are used to reduce error in the measurements; however, thresholds can be altered or removed when data are downloaded.

### Management of data

Respiratory gas exchange data were initially downloaded using standard threshold settings. In this unaveraged format, there were periods of missing data; it was hypothesized that these measurements were outwith the threshold settings and therefore excluded. Data were additionally downloaded with no threshold settings applied to allow comparison. Data were time averaged into 10-s intervals, to reduce variability and random error [21]. Time averaging data reduced the number of missing data points. The remaining missing data with the standard threshold settings were imputed from the data with no threshold settings, which had no missing data points.

Oxygen uptake (mL/kg/min) was extracted for each activity, RER during the graded exercise test, and $\dot{\mathrm{V}}{\mathrm{CO}}_2$ (mL/min) during the measurement of REE. As steady-state measurements provide a more valid representation of the respiratory and metabolic requirements of activity, only minutes 2–4 for each activity was included in the analysis [22]. The final minute of data was excluded from the analysis as some participants became fatigued and agitated towards the end of the 5-min measurement period.

Accelerometer data were sampled at a rate of 30 Hz and was post-processed and reduced to 10-s epochs of data. This duration of epoch was chosen because of the intermittent movements used within the free-living activities, where a shorter epoch will more accurately capture sporadic activity [23]. Data were downloaded into Excel where count data for all activities and measures was extracted. As vertical axis and vector magnitude data are used for calibration, only these measurements were included in the analysis. Accelerometer data were then time matched to the corresponding 10-s epoch of respiratory data. Accelerometer and $\dot{\mathrm{V}}{\mathrm{O}}_2$ data were organized for total activity, with $\dot{\mathrm{V}}{\mathrm{O}}_2$ data additionally organized for each individual participant. Accelerometer data are presented as counts per 10-s epoch (counts/10-s).

### Statistical analysis

All statistical data were analysed using SPSS 21 IBM statistical package (SPSS IBM, New York, NY, USA). Normality was assessed for all variables. For data that were not normally distributed, logarithmic and square root transformations were separately applied to the data and normality was retested. If transformations were not effective in producing normally distributed data, non-parametric tests were used.

Descriptive statistics were calculated for age, sex, height, weight, and body mass index (BMI), with means, standard deviations (SD), and 95 % confidence intervals (95 % CI) reported. Additionally, independent two-sample *t* tests were used to compare differences in these variables between ID and TD participants. The feasibility of activities and REE was assessed from observations and percentage completion rates. The attainment of steady state, defined as a coefficient of variation <10 %, was additionally used to test the feasibility of measuring REE. The feasibility of the graded exercise test was based on attainment of a ${{\dot{\mathrm{V}}\mathrm{O}}_2}_{\max }$ score, with differences between ID and TD participants investigated using independent two-sample *t* tests. The effect of threshold settings was investigated using dependent two-sample *t* tests or the Wilcoxon signed-rank test for data that were not normally distributed. The relationship between $\dot{\mathrm{V}}{\mathrm{O}}_2$ and counts was investigated using Spearman’s correlation coefficients and *z*-scores.

## Results

### Participant characteristics

Five children with mild to moderate ID (four males, one female) and five TD children (one male, four females) participated in this study. Descriptive statistics are presented in Table 2.

**Table 2:** Descriptive statistics for all participants

Description	ID	TD	95 % CI
Age (years)	10.20 ± 1.10	12.40 ± 1.12*	−3.83, −.57
Weight (kg)	36.60 ± 8.08	47.52 ± 5.78*	−21.16, −.68
Height (m)	1.45 ± .08	1.60 ± .09*	−.27, −.03
BMI (kg/m^2^)	17.34 ± 2.85	18.68 ± 2.50	−5.24, 2.57

Data are presented as means, standard deviations, and 95 % confidence intervals for between-group differences

*Significantly (*p* < .05) different from participants with ID

### Recruitment

Seventy-eight children with ID met the inclusion criteria and received an information pack. Ten (12.82 %) initial consent forms were returned, which resulted in a final participation rate of 6 %. Of the parents who returned consent forms but whose child did not participate, reasons given were the need to travel to the laboratory and insufficient time to organize two sessions.

### Activities and measures

#### Resting energy expenditure

Four participants completed the REE measurement for 15 min. One participant became agitated due to wearing the mask and did not complete the measurement. No participant achieved a steady state. The mean coefficient of variation for $\dot{\mathrm{V}}{\mathrm{O}}_2$ and $\dot{\mathrm{V}}{\mathrm{CO}}_2$ for the final 10 min was 24.38 and 28.61 %, respectively. As illustrated by the higher mean $\dot{\mathrm{V}}{\mathrm{CO}}_2$ scores compared to $\dot{\mathrm{V}}{\mathrm{O}}_2$, this measurement caused participants to hyperventilate.

#### Treadmill-based activities

Activity completion rates varied greatly between ID and TD participants. Only one participant with ID completed all activities for the required time. In comparison, all TD participants completed the protocol. The physical demands of the treadmill speeds prevented participants with ID from attempting or completing activities.

Feedback from participants with ID was that the treadmill-based activities were most enjoyable. An interesting observation was the views of parents’, who were present throughout, in relation to their children’s ability to complete the treadmill-based activities. In general, parents underestimated the competence and ability of their child to complete activities. For example, one parent suggested that her child did not participate in the 6 or 8 km/h activities as she had never seen him run and assumed he was not capable of doing so; however, this participant (ID2) completed the 6 km/h activity for 5 min and 8 km/h for 3.5 min.

#### Free-living activities

Three participants with ID completed all free-living activities. Two participants with ID opted out of activities which they did not perceive to be enjoyable. Table 3 shows the activities completed and not completed by participants with ID.

**Table 3:** Activities completed by each participant with intellectual disabilities

Activities and measures	ID 1	ID 2	ID 3	ID 4	ID 5
Session 1					
REE	-	-	-	X	-
Treadmill activities					
3 km/h at 0 %	-	-	-	-	-
6 km/h at 0 %	-	-	-		X
5 km/h at 5 %	-	-	-	-	X
8 km/h at 0 %	-	X	-	X	X
Session 2					
Free-living activities					
Sitting playing DS	-	-	-	-	-
Watching DVD	-	-	-	-	-
Drawing	X	X	-	-	-
Passing football	-	-	-	-	-
Throw/catch	-	X	-	-	-
Standing playing DS	-	-	-	-	-
Step aerobics	-	-	-	-	-
Hula hoop	-	X	-	-	-
Xbox	-	X	-	-	-
Jumping jacks	-	X	-	-	-
Graded exercise test	-	X	-	-	-

- participant completed the activity for the required 5 min, X participant did not complete the activity

### Graded exercise test

Four participants with ID performed the graded exercise test, and one participant opted out due to fatigue. All TD participants performed the test, however, due to a system error, no respiratory gas exchange measurements were recorded for TD1. Individual test data are presented in Table 4. No participant met the primary criteria of a plateau in $\dot{\mathrm{V}}{\mathrm{O}}_2$; therefore, results are presented as the peak scores attained during the test. There were no significant differences between ID and TD participants for $\dot{\mathrm{V}}{\mathrm{O}}_2$ (mL/kg/min), *t*(6) = 1.30, *p* > .05; 95 % CI, −14.67, 4.52, HR (bpm), *t*(7) = −1.61, *p* > .05; 95 % CI, −50.67, 9.67, or RER, *t*(6) = −1.08, *p* > .05; 95 % CI, −.25, .98.

**Table 4:** Individual scores attained during graded exercise test

Participant	${{\dot{\mathrm{V}}\mathrm{O}}_2}_{\mathrm{peak}}$ (mL/kg/min)	HR_peak_ (bpm)	Age-predicted HR_max_ (bpm)	RER_peak_	Speed (km/h)	Time (min)
ID1	34.30	195	209	1.06	5.50	21.50
ID3	30.40	172	209	0.92	5.00	9.00
ID4	25.70	152	211	0.97	4.00	16.00
ID5	31.60	135	211	0.89	3.50	7.00
TD 1	-	177	208	-	6.00	20.50
TD 2	29.40	172	209	1.04	5.50	15.50
TD 3	43.80	199	206	1.21	6.00	14.00
TD 4	30.20	179	208	0.96	6.00	12.00
TD 5	38.90	193	207	0.94	5.50	14.00
ID mean	30.50	163.50	215.00	0.96	4.50	13.38
(SD)	(3.59)	(25.88)	(6.93)	(.07)	(.91)	(6.65)
TD mean	35.57	184.00	207.60	1.04	5.80*	15.20
(SD)	(6.97)	(11.45)	(1.14)	(.12)	(.27)	(3.21)

*TD participants significantly (*p* < .05) different from ID participants

### Breath-by-breath respiratory gas exchange

Usability issues were identified with the respiratory gas exchange equipment. This was due to participant anxiety and the weight of the mask when the bidirectional flow meter and sampling line were attached. All participants expressed anxiety about wearing the mask, although only during the longer duration measure of REE did anxiety affect one participant’s ability to complete the activity. The level of anxiety experienced due to the mask was high; three participants recorded respiratory exchange ratios greater than one, indicating hyperventilation, and one became very upset. Methods employed to reassure participants who were experiencing higher levels of anxiety was a researcher talking to them and a researcher also wearing a mask. However, reported anxiety caused by the mask reduced as the session progressed.

During dynamic movements, the weight of the breath-by-breath valve attached to the mask caused it to slip down, leaving the nose and mouth partially uncovered. All participants were asked to wear a nose clip to limit this effect on the amount of expired gas captured, but no participant agreed to the nose clip. To prevent the mask coming off or slipping off the nose, a researcher held the sample line to reduce the weight the mask had to support. The preVent (Medical Graphics, MN, USA) mask used was the smallest size, and no alternative masks were suitable.

Investigation into the effect of threshold settings showed that $\dot{\mathrm{V}}{\mathrm{O}}_2$ was significantly higher when no thresholds were applied for both ID (*z* = −12.43, *p* < .001, *r* = −.27) and TD participants (*z* = −4.29, *p* < .001, *r* = −.09). Furthermore, when the effect of threshold settings was examined on an individual level, ID participants had a greater variance than TD participants, with percentage change scores ranging from −7.63 to 14.61 % and − .39 to .74 %, respectively.

### Accelerometry and $\dot{\mathrm{V}}{\mathrm{O}}_2$

There was a significant difference in the relationship between $\dot{\mathrm{V}}{\mathrm{O}}_2$ and vertical axis counts (*z* = 13.21, *p* < .0001) and $\dot{\mathrm{V}}{\mathrm{O}}_2$ and vector magnitude counts (*z* = 14.23, *p* < .0001) between ID and TD participants. For participants with ID, $\dot{\mathrm{V}}{\mathrm{O}}_2$ (mean = 11.64, SD = 7.93 mL/kg/min) was significantly correlated with vertical axis (mean = 200.45, SD = 374.35 counts/10 s; *r*_s_ = .70, *p* < .001) and vector magnitude counts (mean = 328.42, SD = 433.45 counts/10 s; *r*_s_ = .73, *p* < .001), with no significant differences between these correlation coefficients (*z* = −1.79, *p* > .05). Similarly, $\dot{\mathrm{V}}{\mathrm{O}}_2$ (mean = 13.89, SD = 8.41 mL/kg/min) was significantly correlated with both vertical axis (mean = 334.25, SD = 529.47 counts/10 s; *r*_s_ = .29, *p* < .001) and vector magnitude counts (mean = 479.20, SD = 569.18 counts/10 s; *r*_s_ = .31, *p* < .001) for TD participants, with no significant differences between correlation coefficients (*z* = .59, *p* > .05).

## Discussion

The purpose of this study was to investigate the feasibility of a laboratory-based accelerometer calibration protocol in children with ID. This section will discuss the results in relation to the four feasibility aims of this study.

### Recruitment

Our initial aim was to recruit ten participants to both the ID and TD groups. Although school-based recruitment strategies have been effective for recruiting TD children to exercise-related studies [24], the school-based strategy used within this study was ineffective, with low response and recruitment rates. The initial response rate of 12.82 % is notably lower than the 83 % response rate reported by Oortwijn and colleagues [16]. There is limited research relating to effective recruitment strategies for children with ID; however, for adults with ID, recruitment strategies involving direct contact with participants are most effective [25]. Furthermore, recruitment of adults with ID is lower for studies involving more invasive measures and physical tests [26].

Small sample sizes and an over-representation of boys are common limitations in health-related research involving children with ID [27]. The over-representation of boys within this study could be partially attributed to the higher prevalence of ID in boys compared to girls [28]. Additionally, as boys generally participate in more physical activity than girls, the activity-focussed protocol may have been of less interest to girls, which could have further limited their recruitment. Another important consideration for the low recruitment rate is the time and travel demands of the study, which were noted by parents as factors which prevented participation. The development of a shorter, single-session protocol would reduce the time requirements of participation, which could subsequently benefit recruitment; however, it is important to consider the effect this could have on participants, such as levels of anxiety and fatigue, and the influence this could have on the quality of data collected.

Despite these recruitment difficulties, it is important that pilot testing is conducted, even with smaller sample sizes, to ensure the feasibility of protocols and measures in this population [29]. Although five participants is not a suitable number for a calibration study, this sample size can still provide meaningful findings relating to the feasibility of calibration protocols and measurements [16]. In future studies, the low response rate needs to be accounted for, although the inclusion of a greater number schools and service organizations could provide the required number of participants for a full-scale calibration study. It is also important to increase our understanding of why girls with ID are frequently underrepresented and to develop girl-focussed recruitment strategies. However, as invasive measures and physical tests are a barrier for adults with ID [26], further investigation is needed to determine whether this low recruitment rate was a direct result of an ineffective recruitment strategy or whether this type of study is one in which children and specifically girls with ID did not want to participate.

### Activities and measures

#### Resting energy expenditure

REE was the first physiological measurement conducted within this protocol. Although participants were given a practice time using the respiratory gas exchange equipment during the preparation phase, wearing the mask for this extended measurement caused increased anxiety and hyperventilation. This affected the attainment of a steady state, which optimizes results and is primarily important for resting metabolic measures [30]. REE is required for deriving metabolic equivalent (MET), therefore necessary in studies aiming to derive prediction equations for activity energy expenditure. However, studies aiming to calibrate intensity cut points do not require a measure of REE, although Freedson et al. [6] noted that activity METs should still be presented in all calibration studies. REE can be approximated through age-specific estimates, therefore, a direct measurement is not essential for the estimation of METs. Based on the difficulties identified within this study for the direct measurement of REE in children with ID, the use of age-specific estimates is deemed most appropriate.

#### Treadmill-based activities

The treadmill speeds were not appropriate for this sample, as the physical demands were too high to allow completion of 5 min for all activities. Previous studies have aimed to ensure the suitability of activities by proposing speeds per age group or within a range of speeds. Puyau et al. [15] included vigorous activities that were age specific; furthermore, Puyau et al. [31] proposed moderate and vigorous activities within speeds of 3.5–4 mph and 4.5–7 mph, respectively. The moderate (5 km/h at 5 % and 6 km/h) and vigorous (8 km/h) speeds in this current study are within the lower ranges used within Puyau et al. [15] and nearest to the vigorous speed for 8–10 years within Puyau et al. [31]. The high completion rate for TD participants could therefore be due to age, as they were significantly older than ID participants. However, three ID participants were still unable to complete the treadmill speeds which were deemed age appropriate in these previous studies, suggesting slower speeds should be used within this population. Employing a range of speeds or age-specific speeds for children with ID could therefore increase the rate of completion.

#### Free-living activities

In contrast, generalizing free-living activities to a calibration study involving children with ID may be more appropriate. The higher completion rates for the free-living activities could be partially due to the intensity not being fixed, as in the treadmill-based activities. Participants were able to complete activities at an intensity that was comfortable for them and could intermittently stop when fatigued. Although this could have a positive effect on completion, it is important that activities are at least semi-structured to ensure participants reach the desired intensity for the purposes of calibration. Due to the limited time that children spend in moderate and vigorous intensity during free play, the use of unstructured activities could negatively impact on the calibration of higher intensity activity [29, 32].

#### Graded exercise test

No participant with ID reached ${{\dot{\mathrm{V}}\mathrm{O}}_2}_{\max }$, which could be due to a number of factors. Firstly, test duration ranged from 7–21.5 min. It is suggested that an exercise test should be completed within 8–12 min to prevent premature termination of the test due to localized muscle fatigue, rather than the attainment of ${{\dot{\mathrm{V}}\mathrm{O}}_2}_{\max }$ [33]. Additionally, the protocol of incrementally increasing gradient can cause calf muscle and lower back discomfort, which limits the participant’s ability to continue with a test [34]. From observation, as the gradient increased, participants became unstable which may have further contributed to the termination of the test before ${{\dot{\mathrm{V}}\mathrm{O}}_2}_{\max }$.

The attainment of ${{\dot{\mathrm{V}}\mathrm{O}}_2}_{\max }$ can be difficult in children who have no prior experience of strenuous exercise and the physical effects and discomfort associated with an exercise test [35]. Furthermore, as none of the sample in this study had prior experience of a treadmill, an alternative test could limit this effect. Field-based tests, including the 1-mile walk test [36], 600-yard walk/run test and 20-m and modified 16-m shuttle run test [37], have shown reliability and concurrent validity in children with ID. These tests could therefore be considered within a calibration protocol as an alternative to a maximal test.

### Breath-by-breath respiratory gas exchange

Measurement issues were identified with the use of the Ultima CPX breath-by-breath system (Medical Graphics, MN, USA), specifically in relation to the use of thresholds. As $\dot{\mathrm{V}}{\mathrm{O}}_2$ is a criterion measure, it is essential that this measurement is accurate to ensure the quality of calibration and prevent systematic error. However, there is currently no universally accepted method for processing breath-by-breath $\dot{\mathrm{V}}{\mathrm{O}}_2$ data, which is impacting on the validity of data processing and interpretation [21]. Freedson et al. [6] discussed the complexity and difficulties associated with using and interpreting a biological criterion measure in children and suggested that a behavioural measure, specifically direct observation, was an effective alternative criterion measure.

Generalizing cut points is partially based on the assumption that the relationship between $\dot{\mathrm{V}}{\mathrm{O}}_2$ and accelerometer counts is the same between groups. However, a significant (*p* < .0001) difference in the relationship of counts and $\dot{\mathrm{V}}{\mathrm{O}}_2$ between ID and TD participants was identified in this study. Therefore, the prediction of intensity classification for ID children based on TD cut points will introduce systematic error and validity issues. Although the validity of generalizing cut points between TD and ID children has been previously discussed [7], this is the first study which aimed to compare the fundamental relationship between $\dot{\mathrm{V}}{\mathrm{O}}_2$ and counts. This further supports the need for a calibration study to be conducted specifically in a population of children with ID; however, the feasibility issues identified need further consideration before a full-scale laboratory-based calibration study.

### Strengths and limitations

This was the first study which aimed to address the lack of population-specific cut points for children with ID. Furthermore, to ensure the suitability and effectiveness of a gold standard laboratory-based protocol, this study investigated the feasibility of activities and measurements. The wide range of treadmill and free-living activities included within this study increased our knowledge relating to the appropriateness of generalizing activities used with TD children to children with ID. The findings from this study can therefore be used to inform the development of an appropriate protocol.

The design of the present protocol had an effect on completion rates and data collection, as participants became fatigued during the latter stages of the session, in particular the second session. Therefore, the conclusions regarding the appropriateness of activities could be affected by study design. It is also important to note that as all hypothesis testing is based on a small sample size, results should be interpreted with caution. Although the small sample size highlights the difficulties with recruitment, it prevented direct comparison with a group of matched TD participants which would have enabled further investigation into physiological differences. Furthermore, as no in-depth data was collected regarding the aetiology of ID, it is not possible to discuss possible effects of ID type or severity on participation and activity completion rates.

## Conclusions

Findings from this study suggest that the methods used within a calibration protocol for TD children cannot be fully generalized to children with ID. Although additional research is required before definitive conclusions can be made regarding feasibility, initial methodological recommendations for the design of a calibration study involving children with ID are as follows: 1) Treadmill activities should not be generalized from protocols involving TD children; instead, speeds should be self-selected or age-appropriate speeds developed. 2) Free-living activities, which can be successfully generalized from TD protocols, should be incorporated due to the high completion rates. 3) REE and ${{\dot{\mathrm{V}}\mathrm{O}}_2}_{\max }$ should be estimated using validated non-invasive methods. In terms of future research, it is recommended that the suitability and validity of breath-by-breath respiratory gas exchange measurements is further investigated. Furthermore, an effective recruitment strategy has to be developed, and reasons for the low recruitment rate of girls need to be better understood.

Until the limitations identified within this study have been addressed, the use of a laboratory-based calibration protocol is not feasible for children with ID. As these limitations are specific to a laboratory-based protocol, consideration should therefore be given to an alternative protocol. A field-based calibration study which is conducted in the participants’ environment, e.g. school, and which uses a non-invasive criterion measure, such as direct observation, could be an effective alternative to a laboratory-based study.

## Appendix

**Table 5:** Activity protocols of previous child calibration studies which included treadmill and free-living activities

Study	Participant age range (years)	Treadmill activities	Time per treadmill activity (min)	Free-living activities	Time per free-living activity (min)
Eston et al. [13]^a^	8–10	Walk 4 km/h	4	Playing catch	4
		Walk 6 km/h	4	Hopscotch	4
		Run 8 km/h	4	Sitting crayoning	4
		Run 10 km/h	4		
Evenson et al. [14]^b^	6–8	Light		Sedentary	
		Walk 2 mph	7	Rest	15
		Moderate		Sitting watching DVD	7
		Walk 3 mph	7	Sitting colouring books	7
		Vigorous		Moderate	
		Run 4 mph	7	Stair climb	7
				Dribble basketball	7
				Vigorous	
				Cycle on stationary bike	7
				Jumping jacks	7
McMurray et al. [35]^b^	8–18	Walk 4 km/h	10	Standing arcade game	10
		Walk 5.6 km/h	10	Stretching	10
		Run 8 km/h	10	Sweeping	10
				Vacuuming	10
				Shovelling	10
				Stair climb (88 steps/min)	10
				Rope skipping	10
Puyau et al. [15]^c^	6–16	Light		Sedentary	
		Walk 2.5 mph	10	Rest	20
		Moderate		Sitting playing Nintendo	20
		Walk 3.5 mph (6–7 years)	10	Arts and crafts	20
		Walk 4 mph (8–16 years)	10	Free play sitting, e.g. cards, lego, miniature cars	20
		Vigorous		Light	
		Jog 4.5 mph (6–7 years)	10	Aerobic warm up	10
		Jog 5 mph (8–10 years)	10	Moderate	
		Jog 6 mph (11–16 years)	10	Tae Bo martial arts exercises	10
				Free play standing, e.g. hula hoop, throwing ball, jumping jacks	10
				Vigorous	
				Jump rope	3
				Walk—self-selected pace	5
				Skip	3
				Jog—self-selected pace	3
				Soccer	3
Puyau et al. [28]^c^	7–18	Walk 2 mph	7	Rest	30
		Walk 3.5–4 mph	7	Sitting playing handheld Nintendo	20
		Jog/run 4.5–7 mph	7	Sitting playing computer	20
				Cleaning (dusting)	10
				Aerobic exercises	12
				Ball toss	10
Vanhelst et al. [23]^a^	10–16	Light	15 min consecutive	Sedentary	15 min consecutive
		Walk 1.5 km/h, 3 % gradient		Lying in bed watching TV	
		Moderate		Sitting reading	
		Walk 3 km/h, 3 % gradient		Sitting playing computer game	
		Run 4 km/h, 3 % gradient		Light	
				Standing drawing	
		Vigorous		Passing football	
		Run 6 km/h, 3 % gradient			

^a^Respiratory gas exchange was measured using a stationary metabolic system, which restricts movement within a laboratory environment

^b^Respiratory gas exchange was measured using a portable metabolic system, which allows almost unrestricted movement within a designed indoor or outdoor area

^c^Respiratory gas exchange was measured using a whole room calorimeter, which allows unrestricted body movement within a confined room
